# Full-color dynamic volumetric displays with tunable upconversion emission from RE^3+^-doped glasses (RE = Ho, Tm, Nd, Yb) under NIR laser excitation

**DOI:** 10.1038/s41377-024-01672-2

**Published:** 2025-01-02

**Authors:** Utku Ekim, Diğdem Özkutay, Miray Çelikbilek Ersundu, Ali Erçin Ersundu

**Affiliations:** Yildiz Technical University, Faculty of Chemical and Metallurgical Engineering, Department of Metallurgical and Materials Engineering, Glass Research and Development Laboratory, Istanbul, 34220 Türkiye

**Keywords:** Displays, Photonic devices

## Abstract

Three-dimensional (3D) imaging technology holds immense potential across various high-tech applications; however, current display technologies are hindered by limitations such as restricted viewing angles, cumbersome headgear, and limited multi-user accessibility. To address these challenges, researchers are actively exploring new materials and techniques for 3D imaging. Laser-based volumetric displays (VDs) offer a promising solution; nonetheless, existing screen materials fall short in meeting key requirements for long-term durability, full-color operation, and scalability. In this study, we present a comprehensive investigation into easily scalable rare-earth (RE^3+^) doped monolithic glasses (RE = Ho, Tm, Nd, Yb) capable of tunable full-color emission using a novel excitation modulation technique under 808 nm and 980 nm laser excitation and demonstrate their implementation as laser-based VD materials through prototyping. By controlling the movement of lasers’ pulses and galvanometer mirrors with waveform generators, our system generates images in simple and complex shapes with high purity red, green, and blue (RGB) colors. These images can be manipulated, including actions like translation, rotation, expansion, and sequential movement within the monolithic glass screen material. Our findings showcase the potential of glass-based dynamic VDs in revolutionizing display technology, offering superior color purity, vividness, and performance in comparison to conventional display systems.

## Abstract (web summary)

Rare-earth-doped glasses with tunable full-color emission under NIR laser excitation enable scalable, and high-performance dynamic volumetric displays, offering superior RGB purity and shape control for advanced 3D imaging applications.

## Graphical abstract

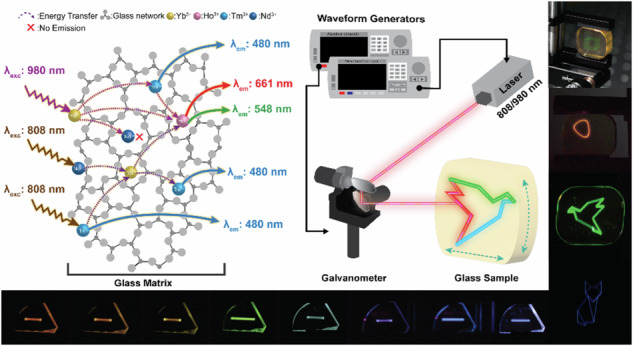

## Introduction

Human stereoscopic vision excels in perceiving depth in a three-dimensional (3D) world, yet modern display technologies are predominantly two-dimensional (2D), which limits the depth information available and thereby restricts our visual perception capabilities^1^. In response, technologies such as head-mounted displays (virtual reality) and stereoscopic displays have been developed and commercialized^2^. These systems convert 3D visuals into 2D images and then reconstruct them into 3D, often resulting in less natural visual experiences^3^. They also face several drawbacks, including a limited field of view^4^, discomfort from headgear that can cause eye fatigue^5^, single-user accessibility, and high computing power requirements^3^. Volumetric displays (VDs) offer a promising alternative by generating true 3D images using volumetric pixels (voxels), which allows for enhanced field of view, spatial resolution, and dynamic imagery without relying on wearables or visual tricks^6^. This innovation results in a superior field of view, spatial resolution, dynamic imagery, and ease of application compared to existing virtual reality, stereoscopic, and even holographic display systems^3,6–8^. Although reflection (scattering)-based VDs utilizing incoherent light sources are currently available, they suffer from issues like restricted viewing angles, limited depth perception, challenges in content creation, and low resolution^4^. Laser-based VDs, though not yet commercially available, offer significant advantages such as vivid colors, high contrast ratios, and a wider color gamut due to the inherent properties of lasers^9,10^. Despite these benefits, there are still key challenges that need to be addressed before laser-based VDs can be effectively integrated into practical applications in fields such as medicine, engineering, architecture, education, and military^6,11^.

Previous laser-based VD approaches reported the use of different kinds of screen materials, each with its advantages and limitations. Downing et al. pioneered 3D VDs using upconverting rare-earth (RE^3+^) doped glasses^12^. Due to their isotropic nature, glasses do not impose polarization limitations on pump lasers, ensuring isotropic light emission from every voxel. However, their method was inefficient due to the need for multiple glass layers to achieve red, green, and blue (RGB) colors, resulting in challenges like low resolution and ghost voxels due to varying screen material responsiveness under different wavelength irradiation. Other attempts at laser-based VDs using glasses excited in the up-conversion regime include non-linear optical crystal dispersed glass ceramics^9^ and RE^3+^-doped monolithic glasses^5,13,14^. Non-linear optical crystal dispersed glass ceramics produce primary-colored emissions under triple beam excitation^9^ but require high optical power and femtosecond pulsed lasers for photoluminescence (PL) via second harmonic generation, leading to voxel size complications due to crystallite size, which restrict spatial resolution and cause voxel size inconsistencies. In the case of other RE^3+^-doped monolithic glasses, excitation by traditional continuous wavelength (CW) irradiation addresses manufacturing method complications but sacrifices color tunability, rendering them monochromatic^5,13,14^. Apart from glasses, Gu et al.^15^ and Wan et al.^16^ utilized photo-activated phosphorescence of porphyrins in dimethyl sulfoxide (DMSO) solution within a quartz cube to create 2D and 3D structures. However, improvements are needed for light emission durations, contrast, and polychromatic images. Patel et al.^6^ demonstrated 2D and 3D-like images within a quartz cube using photoactivatable dyes, but they were limited to single-color images due to dye durability constraints. Recently, there has been growing interest in RE^3+^-doped upconversion nanoparticles (UCNPs) due to their ability to be excited by cost-effective steady-state lasers^8,17–23^. This capability enables the generation of orthogonal RGB color images using core-shell nanostructures. Wang et al.^24^ reported the incorporation of NaYF_4_:Yb/Er/Gd UCNPs into PDMS monoliths for volumetric 3D displays, using a 980 nm diode laser in a single-beam design to address ghost voxel and alignment issues. Although this work was pioneering at the time, it was limited to generating only green images and did not explore the potential for creating full RGB color displays, leaving room for further advancements. Later, Jia et al.^25^ proposed dynamic multicolor emissions from a single activator Er^3+^ ion within single NaYF_4_ UCNPs under tunable synergistic excitation with 980/1973 nm NIR light. Nevertheless, the absence of blue emission, along with the limited availability of 1973 nm lasers and the poor stability of epoxy resin used for imaging, hinders broader adoption. Zhao et al.^26^ developed LiYO_2_:RE^3+^/Yb^3+^ (RE = Tm, Ho, Eu) phosphors and their 3D printed patterns in PDMS optimized for tricolor upconversion luminescence under 980 nm laser excitation. Despite these advancements, challenges with static images and stability still need to be addressed. In summary, creating core-shell nanostructures demands meticulous design and precise control over the synthesis process. As the number of layers increases, the experiment becomes increasingly intricate and challenging to replicate. Additionally, multi-color emitting core-shell nanostructures often encounter adverse effects like energy migration, re-absorption, and self-filtering, which compromise color purity and limit the achievable color gamut^22^.

Despite the advantages and disadvantages of previous materials, the search for alternative methods and materials for fully functional and colorful laser-based VDs with simplified fabrication, controlled scalability, and higher color purity persists, underscoring the significant challenge of controlling emission color across a broad range of wavelengths within a single material. At this point, special glasses emerge as promising VD materials compared to crystalline material hosts due to outstanding properties such as, high optical transmittance, high thermal and chemical stability, high RE^3+^ solubility, high optical damage threshold, low phonon energy that enables the enhancement of up-conversion emission, ease of large-scale production and superior mechanical resistance^27^. Herein, building on our prior research^28^, we present a comprehensive investigation into specially designed upconverting RE^3+^-doped monolithic glasses (RE = Ho, Tm, Nd, Yb), capable of tunable full-color emission using a novel excitation modulation technique under 808 nm and 980 nm laser excitation, and demonstrate their implementation as laser-based VD materials through prototyping. In our previous work^28^, the blue emission achieved with Tm^3+^ ions alone was found to be insufficient. In this study, we address this limitation by incorporating Nd^3+^ ions, which significantly enhances the blue emission. The combination of Nd^3+^ (serving as a sensitizer at 808 nm excitation) with Yb^3+^ (acting as a sensitizer at 980 nm excitation and an activator at 808 nm excitation) allows us to extend the range of excitation wavelengths and improve emission efficiency across three primary colors. This approach also involves careful optimization of Nd^3+^ concentration to prevent undesirable low-intensity transitions, thereby achieving a more balanced and efficient emission spectrum. By integrating these two excitation sources into a single monolithic glass, we generate a diverse range of basic and intricate static and dynamic visualizations, showcasing a broad spectrum of RGB colors. The prototypes presented here highlight the significant potential of these specialized glasses as screen materials for laser-based VDs.

## Results

### Optical absorption and PL properties

**Fig. 1:**
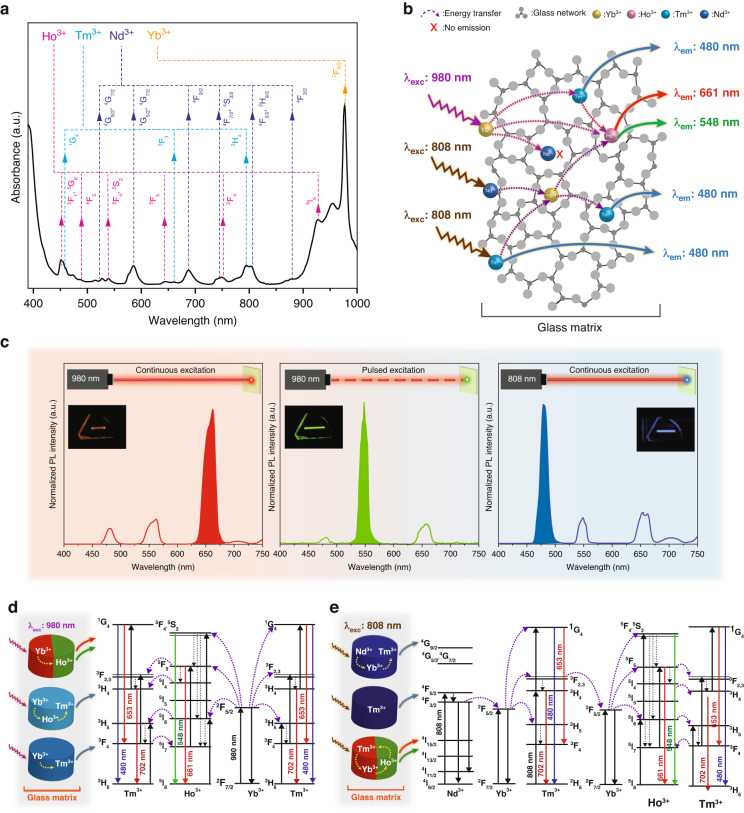
**a** Optical absorption spectrum showing all possible transitions for Ho^3+^, Tm^3+^, Nd^3+^ and Yb^3+^ ions. **b** Schematic illustration of the RE^3+^-doped glass structure for RGB luminescence under 808 and 980 nm excitation. **c** Normalized PL emission spectra under continuous 980 nm (left), 980 nm pulsed (middle), and continuous 808 nm (right) excitation. (The insets show the corresponding luminescence photographs). Schematic illustrations of the luminescence mechanism and proposed excited-state level population pathways for producing tunable multicolor emissions under **d** 980 nm and **e** 808 nm excitations

Figure 1a illustrates the optical absorption spectrum of glass samples doped with RE^3+^ ions (0.05%Ho_2_O_3_-0.25%Tm_2_O_3_-0.05%Nd_2_O_3_-2%Yb_2_O_3,_ in mol%). The spectrum reveals sixteen distinct absorption peaks, including seven centered at 452, 487, 538, 643, 750 and 927 nm, corresponding to transitions of Ho^3+^ starting from the ground state ^5^I_8_ to excited levels ^5^F_1_-^5^G_6_, ^5^F_3_, ^5^F_4_-^5^S_2_, ^5^F_5_, ^5^F_4_ and ^5^I_5,_ respectively^29,30^. Furthermore, the bands located at 458, 661, and 794 nm correspond to the transitions from the ^1^G_4_, ^3^F_3_, and ^3^H_4_ excited states of Tm^3+^, respectively^31^.

However, the absence of the ^3^H_6_ and ^3^F_4_ bands, typically observed for Ho^3+^ ion around 360 and 374 nm, is attributed to the increased absorbance of the glass near its absorption edge at approximately 420 nm. Additionally, six distinct bands corresponding to the ^4^G_9/2_-^4^G_7/2_, ^4^G_5/2_-^2^G_7/2_, ^4^F_9/2_, ^4^F_7/2_-^4^S_3/2_, ^4^F_5/2_-^2^H_9/2_, and ^4^F_3/2_ transitions of Nd^3+^ are located at 527, 586, 688, 747, 805 and 875 nm, respectively^32^. The Yb^3+^ ions exhibit a unique and strong absorption band corresponding to ^2^F_5/2_, widely aroused around 980 nm^27^. Consequently, the optical absorption spectrum demonstrates the feasibility of exciting RE^3+^-doped glass samples using dual-channel pumping.

Figure 1b schematically depicts the structure of the RE^3+^-doped glass. Each RE^3+^ ion in the glass matrix contributes uniquely to the upconversion process, collectively resulting in the generation of RGB emissions. Yb^3+^ and Nd^3+^ ions serve as effective sensitizers for capturing excitation at 980 nm and 808 nm, respectively. Yb^3+^ ions here play a crucial role by absorbing 980 nm incident photons and transferring energy to the main radiative energy levels of neighboring Tm^3+^ and Ho^3+^ ions. This mechanism induces blue emissions and weaker red emissions (ignored in Fig. 1b) from Tm^3+^ ions, alongside dominant green and red emissions from Ho^3+^ ions. On the other hand, the purity of blue emission is further enhanced by Tm^3+^ ions alone under 808 nm excitation (Fig. S1a), along with the energy transfer pathways of Nd^3+^→Yb^3+^→Tm^3+^. Additionally, weaker red and green emissions are observed due to energy transfer pathways from Tm^3+^→Yb^3+^→ Ho^3+^ under 808 nm excitation (Fig. S1b).

Figure 1c presents the PL spectra of the RE^3+^-doped glass sample under CW and pulsed excitation at 980 nm, and CW excitation at 808 nm, respectively. The spectra exhibit distinct emissions: a 661 nm (red) emission is observed under both CW and pulsed excitation at 980 nm, while a 548 nm (green) emission is predominantly obtained with pulsed excitation at 980 nm. Additionally, a dominant 480 nm (blue) emission is generated exclusively under CW excitation at 808 nm. However, the relative intensities of the RGB peaks vary depending on the excitation modulation, which involves adjusting the frequency and pulse width parameters together with power density.

Figure 1d depicts the energy level diagrams and proposed UC mechanisms of Ho^3+^, Tm^3+^, Nd^3+^ and Yb^3+^ ions in the glass sample under 980 nm and 808 nm excitations, enabling precise color-tunability. Various energy transfer processes and emissions occur under these excitations. When excited by the 980 nm laser, Yb^3+^ ions transition from the ^2^F_7/2_ state to the ^2^F_5/2_ state, acting as sensitizers and transferring the absorbed excitation energy to adjacent Ho^3+^ and Tm^3+^ ions. Excited Yb^3+^ ions transfer energy to Ho^3+^ ions in their ^5^I_6_, ^5^F_5_, and ^5^S_2_, ^5^F_4_ energy states. For the 661 nm red emission, Ho^3+^ ions are excited from their ground ^5^I_8_ state to ^5^I_6_ and ^5^S_2_, ^5^F_4_ states, followed by non-radiative relaxation to the ^5^F_5_ state, emitting excess energy. Alternatively, excitation of the ^5^I_6_ energy state of Ho^3+^ ions can also lead to the excitation of the ^5^F_5_ and ^5^I_7_ energy levels through non-radiative relaxations, resulting in the same red emission from Ho^3+^ ions. Additionally, excitation of the ^5^I_6_ energy state of Ho^3+^ ions, following various non-radiative relaxations, can lead to the excitation of the ^3^H_5_ and ^3^H_4_ energy states of Tm^3+^ ions via energy transfer, ultimately resulting in blue and red emissions. On the other hand, Ho^3+^ ions excited from their ground state to ^5^F_4_, ^5^S_2_ states release excess energy directly, resulting in 548 nm green emission. Energy transfer from the ^2^F_5/2_ state of Yb^3+^ ions to the ^3^H_5_, ^3^F_2,3_ and ^1^G_4_ energy states of Tm^3+^ ions results in dominant blue and weaker red emissions. This occurs through non-radiative relaxations and transitions, specifically ^1^G_4_ → ^3^H_6_, ^1^G_4_ → ^3^F_4_ and ^3^H_4_ → ^3^H_6_, emitting at 480 nm, 653 nm and 702 nm, respectively. Additionally, excited Ho^3+^ ions transfer energy to the ^3^F_2,3_, ^3^H_4_, and ^3^H_5_ energy states of Tm^3+^ ions via energy transfer and non-radiative mechanisms, enhancing the blue emission. Furthermore, excited Tm^3+^ ions transfer energy back to the ^5^I_7_ energy states of Ho^3+^ ions from their ^5^F_4_ state, boosting the red emission of Ho^3+^ ions. Nd^3+^ ions also undergo radiative transitions due to the excitation of Yb^3+^ ions at 980 nm. However, energy transfers between Yb^3+^ and Nd^3+^ are not depicted in Fig. 1d, as the low-intensity radiative transitions of Nd^3+^ ions are not observable in the visible range (Fig. S2). In summary, the excitation of Yb^3+^ at 980 nm is crucial as it absorbs photons and transfers energy to the ^3^H_5_, ^3^F_2,3_ and ^1^G_4_ states of Tm^3+^ ions and the ^5^I_6_, ^5^F_5_, and ^5^S_2_, ^5^F_4_ states of Ho^3+^ ions, resulting in RGB emissions in the visible region.

Under excitation with an 808 nm laser, Tm^3+^ ions are excited from their ground ^3^H_6_ state to the ^3^H_4_ and ^1^G_4_ states, emitting excess energy predominantly as blue light at 480 nm and weaker red light at 653 nm through the ^1^G_4_ → ^3^H_6_ and ^1^G_4_ → ^3^F_4_ transitions, respectively. It is known that under 808 nm excitation, Tm^3+^ ions also emit blue radiation at 450 nm as a result of the ^1^D_2_ → ^3^F_4_ transition. As already mentioned in the optical absorption spectrum (Fig. 1a), this well-known blue emission of Tm^3+^ ions is not visible due to the increased absorbance of the glass near its absorption edge. In addition to the blue emission resulting from Tm^3+^ ions, Nd^3+^ ions are vital for blue emissions. In contrast to our prior study^28^, where blue emission was inadequate when using Tm^3+^ ions alone, the introduction of Nd^3+^ ions in the glass sample amplifies and triggers blue emissions. The combination of Nd^3+^ and Yb^3+^ ions, serve as a reservoir for the blue emission process under 808 nm excitation acting as sensitizer and activator ions, respectively. When excited by the 808 nm laser, Nd^3+^ ions excited from their ^4^I_9/2_ ground state to ^4^F_5/2_, the excess energy can be transferred to Yb^3+^ ions, exciting their metastable ^2^F_5/2_ state. Energy from the excited Yb^3+^ ions then can be transferred to Tm^3+^ ions, resulting in blue and red emission at 480 nm and 653 nm by ^1^G_4_ → ^3^H_6_ and ^1^G_4_ → ^3^F_4_ transitions, respectively. Briefly, Nd^3+^, Tm^3+^ and Yb^3+^ ions work together to boost ^1^G_4_ level of Tm^3+^ ions, enhancing blue emission by ^1^G_4_ → ^3^H_6_.

In summary, the combined presence of Ho^3+^, Tm^3+^, Nd^3+^ and Yb^3+^ ions enables the absorption of infrared excitation at 980 nm and 808 nm, leading to the emission of energy across the three primary RGB colors in the visible region. This aspect is crucial for laser-based VDs, as infrared emissions are imperceptible to the human eye and do not interfere the desired image formation.

### Excitation modulation and colorimetric analysis

**Fig. 2:**
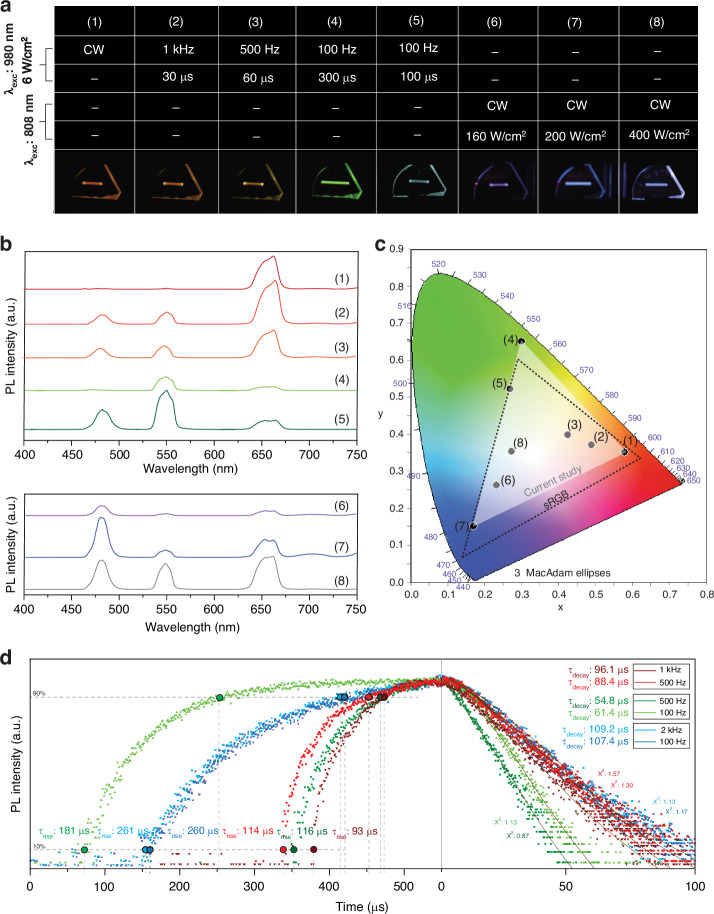
**a** Luminescence photographs of RE^3+^-doped glasses showing multicolor upconversion luminescence tuning (1–8) under varying excitation frequencies and pulse widths with 980 nm and 808 nm laser excitation. **b** Corresponding upconversion PL spectra of the glasses depicted in (**a**). **c** Corresponding CIE chromaticity diagram showing the calculated luminescence colors from the upconversion PL spectra, compared to the sRGB color gamut. **d** Time-resolved PL spectra of the glass sample under different excitation frequencies for RGB emission bands (left: rising profiles, right: decay profiles)

RE^3+^-doped glasses exhibit dynamic multicolor luminescence tuning, ranging from red to green to blue, under 980 nm and 808 nm laser excitation (Fig. 2a). Accordingly, laser sources are utilized alongside a waveform generator, allowing for adjustments in power density, frequency, and pulse width parameters to achieve varied colored emissions. Figure 2b displays the upconversion emission spectra when excited by a 980 nm laser at a constant power density of 6 W/cm^2^ with varying excitation frequencies and pulse widths (Fig. S3), as well as by an 808 nm laser with CW excitation at increasing power densities. We observed that under 980 nm CW excitation, the spectrum is predominantly dominated by red emission. Interestingly, this red emission pattern remains largely unchanged when transitioning to pulsed modulation at 980 nm excitation, while maintaining high duty cycles. However, at lower frequencies, the intensity of red emissions in PL spectra decreases as green emissions increase, resulting in color transitions from red to orange at a frequency of 1 kHz and a pulse width of 30 µs, then to yellow at a frequency of 500 Hz and a pulse width of 60 µs, and finally to green at a frequency of 100 Hz and a pulse width of 300 µs, all while maintaining a constant 3% duty cycle. Subsequently, maintaining a constant frequency of 100 Hz and decreasing the pulse width to 100 µs (with a duty cycle of 1%) leads to cyan emission. However, remarkably, when the excitation wavelength is switched to 808 nm, the blue emission in PL spectra is further intensified by the presence of Tm^3+^ ions, facilitated by the energy transfer pathways of Nd^3+^→Yb^3+^→Tm^3+^. Under 808 nm CW excitation, as the excitation power density gradually increases from 160 W/cm² to 200 W/cm², the emission color transitions from purple to blue. Finally, with a further increase in power density to 400 W/cm², a bluish-white emission is observed (Fig. S4). Figure 2c and Table S1 illustrate the corresponding CIE chromaticity diagram and detailed coordinates, respectively, depicting the calculated luminescence colors from the upconversion PL spectra, and compare them against the sRGB color gamut^33^. The color gamut covered by RE^3+^-doped glasses encompasses 79.88% of the sRGB color gamut.

To the best of our knowledge, this color gamut is slightly higher than that of a previous study using dual-channel pumping for UCNPs with the same laser excitation wavelengths^8^. However, it is somewhat lower than other reported color gamuts based on UCNPs excited by three laser sources^18,19,21,22^. While we acknowledge there is still room for improvement, the obtained color gamut suggests that it is possible to construct full-color images in RE^3+^-doped glasses laser-based VDs.

To understand the mechanism behind tunable upconversion emission via frequency modulation, time-resolved PL measurements of visible emissions at 480 nm (blue), 548 nm (green), and 661 nm (red) are conducted. These measurements are performed under 808 nm excitation (for 480 nm) and 980 nm excitation (for 548 and 661 nm), with a pulse duration of 550 μs and at varying frequencies. The corresponding time-resolved PL spectra, showing both the rising and decay profiles, are depicted in Fig. 2d. As is well known, rise time refers to the duration it takes for the PL signal to reach its peak after excitation, while decay time is the period during which the signal decreases to a fraction of its maximum after the excitation is removed. As evident from the rising profiles, reflecting the varied buildup durations of upconversion luminescence states, distinct population times are discernible under pulsed excitation. The recorded profiles are analyzed, yielding experimental rise times for emissions at 480 nm under 100 Hz and 2 kHz, with values of 260 μs and 261 μs, respectively. For emissions at 548 nm, rise times of 181 μs are recorded under 100 Hz, and 116 μs under 500 Hz. Finally, for emissions at 661 nm, rise times of 114 μs are observed under 500 Hz, and 93 μs under 1 kHz. Under 980 nm excitation, a frequency of 100 Hz is adequate for populating the ^5^F_4_ and ^5^S_2_ green states, while achieving a population of ^5^F_5_ red states necessitates higher frequencies due to the slower energy transfer process involving non-radiative relaxations. Therefore, green emission dominance can be achieved with low-frequency excitation to prevent undesirable energy transfer between the ^5^I_6_ and ^5^I_7_ states. Conversely, high-frequency excitation promotes non-radiative relaxation through the ^5^I_7_ state, resulting in enhanced red emission (Fig. S5). As depicted in Fig. 1d, the ^1^G_4_ blue state undergoes a three-step population process, taking longer due to two non-radiative relaxations. Therefore, as previously noted in our prior study^28^, due to the low population rate of the ^1^G_4_ blue state compared to green and red emitting states, even with short pulses under 980 nm excitation, blue radiation does not exceed other colors, particularly red. As a result, dominance of blue radiation over green and red emission under 980 nm excitation remains unachievable for RE^3+^-doped glasses. However, by switching the excitation wavelength to 808 nm and incorporating Nd^3+^ ions into the glass network, the population of the ^1^G_4_ blue state at both low and high-frequency excitations becomes feasible. This is due to the energy transfer pathways of Nd^3+^→Yb^3+^→Tm^3+^, which facilitate blue emission. Following this, recorded decay profiles are analyzed, yielding experimental lifetimes for emissions at 480, 548, and 661 nm ranging from 107.4 to 109.2 μs, 54.8 to 61.4 μs, and 88.4 to 96.1 μs, respectively, across different frequencies. As expected from the nonlinear upconversion process, the decay lifetimes of each emission are shorter than the rise times. In summary, under 980 nm excitation with short pulse width and relatively low frequency, green emission intensity exceeds that of red emission, whereas high frequency promotes red emission through facilitated non-radiative relaxation. However, this phenomenon does not apply to blue radiation, as altering the frequency does not affect the population of blue states, rendering tuning of blue radiation through frequency modulation infeasible.

### RGB emitting image construction

**Fig. 3:**
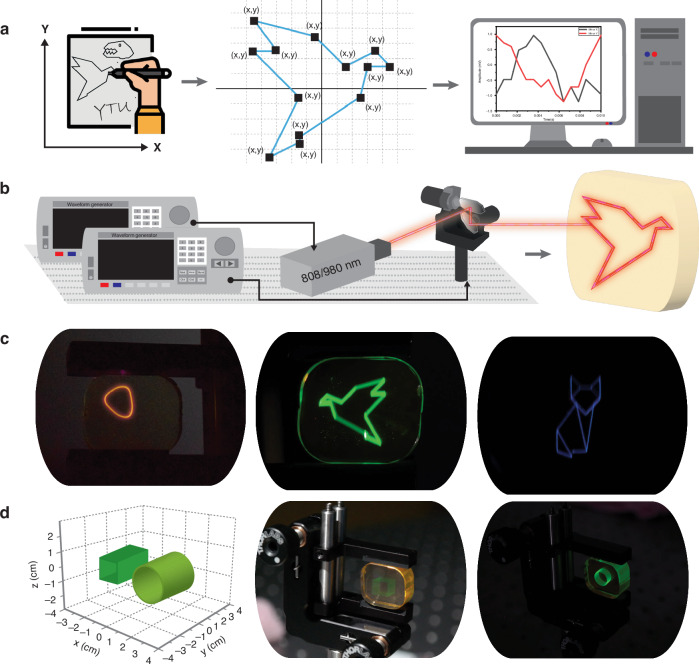
**a** Creation of selected shapes, mapping of the shapes onto a Cartesian coordinate system, and entering of (x, y) points into the computer software. **b** Synchronization of separate mirror signals via pulse modulation using two waveform generators to tailor parameters such as frequency, pulse width, and power density to optimize laser operation, and construction of selected image. **c** Front view photographs depicting the color images emitted from the RE^3+^-doped monolithic glasses (red circle under 980 nm continuous excitation (left), green bird under 980 nm pulsed excitation (middle), blue cat 808 nm continuous excitation (right). **d** Schematic illustration of the computational design process for generating images (left), side views of images (a green cube (middle) and a green cylinder (left)) created in the volume of glass sample under 980 nm pulsed excitation under standard indoor lighting conditions and in darkness, respectively

As a proof of concept, a custom setup utilizing two lasers operating at distinct wavelengths (808 nm and 980 nm) is designed to direct the laser path onto a galvanometer for creating selected images within the glass sample (Fig. S6). For the image construction process, selected shapes are initially mapped onto a Cartesian coordinate system. The coordinate boundaries are selected within the operational range of the galvanometer (±5 V). Each image’s corners or points where movement direction changes are designated as (x, y) points on the plane. The x-values represent the voltage applied to the x-mirror, while the y-values indicate the voltage amplitude for the y-mirror. These determined values serve as the amplitudes of the signals sent to the galvanometer. Subsequently, these points are input into computer software, which determines the optimal function between the points to generate the desired image (Fig. 3a). Separate signals are generated for each mirror, with time adjustments made via pulse modulation to synchronize movement with another waveform generator connected to both lasers. For shapes with discrete regions, laser pulses are altered to pause at appropriate locations. The specifications of the galvanometer and laser signals are tailored with varying parameters such as frequency, pulse width, and power density to construct the selected image, and synchronization is achieved by adjusting the phase difference (Fig. 3b).

Here, we conduct an application demonstration of the volumetric color displays. Figure 3c shows front-view photographs, while Fig. 3d depicts a schematic illustration of the computational design process for generating RGB images, along with their side views, obtained through the interaction of the created shapes with the glass sample using the described setup. All images are captured using a Nikon D3300 camera equipped with a Nikon AF-P DX NIKKOR 18–55 mm lens to absorb any remaining light from the excitation laser.

As evident from the images, the generation of red, green, and blue colored images is enabled using different laser irradiation on the same monolithic glass sample. With precise Cartesian coordinate input, the galvanometer also enables the generation of different intricate images, ranging from basic geometric shapes to complex drawings (Fig. S7).

**Fig. 4:**
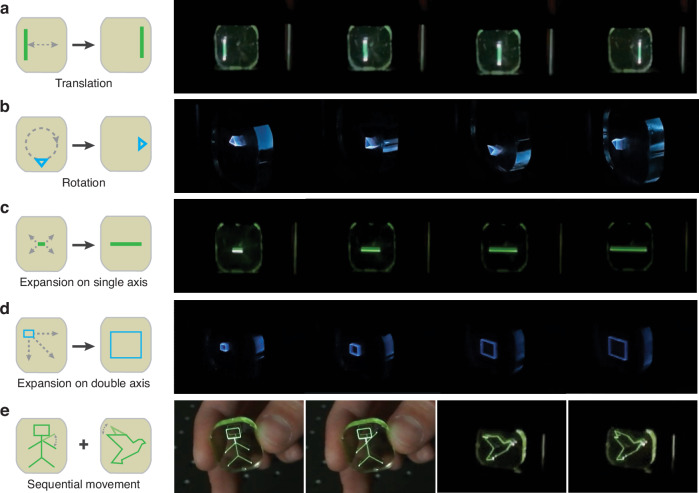
Schematic illustrations and luminescent images of VDs based on RE^3+^-doped monolithic glass, demonstrating (**a**) translation, (**b**) rotation, (**c**) expansion on a single axis, (**d**) expansion on double axis, and (**e**) sequential movement of different images. Green emissions (**a**, **c**, **e**) occur with 980 nm pulsed excitation, while blue emissions (**b**, **d**) result from 808 nm continuous excitation

In addition to static images, the ability to achieve full-color tunability under different excitation parameters allows the same setup to create moving images, which are recorded at speeds of 60 frames per second using a standard cell phone camera. Figure 4a–e and Video S1–S6 showcase luminescent RGB images of selected figures demonstrating: the translation of a green vertical line from left to right, the rotation of a blue triangular prism, the expansion of a green horizontal line on a single axis, the expansion of a blue cube on a double axis, and the sequential movement of a human figure waving alongside a bird figure flapping its wings, respectively.

Green emissions (Fig. 4a, c, e) are observed under 980 nm pulsed excitation, while blue emissions (Fig. 4b, d) are under 808 nm continuous excitation. Unlike previous approaches to volumetric color displays, which typically entail complex multilayer core-shell structures^8,17–23^, our method employs a novel excitation modulation technique under 808 nm and 980 nm laser excitation for multicolor luminescence tuning and image generation utilizing a readily producible and highly scalable monolithic glass material. In summary, we anticipate that our demonstration will significantly advance the commercialization of glass-based VDs, showcasing the technique’s potential for 3D imaging while emphasizing improvements in color accuracy.

## Discussion

In conclusion, we have successfully developed a proof-of-concept VD utilizing monolithic tellurite glass as the host material. By incorporating RE^3+^ (RE = Ho, Tm, Nd, Yb) ions into the glass composition and employing 980 nm and 808 nm laser stimulation sources, we have demonstrated the creation of images in simple and complex shapes in RGB colors within the monolithic glass material. Our methodology, which involves precise control of laser pulses and galvanometer mirrors using waveform generators, enables manipulation of the generated images through translation, rotation, expansion, and sequential movement on the glass screen. Importantly, the unique properties of the glass material allow for the production of displays in various sizes without sacrificing image quality, avoiding potential color and sharpness losses associated with traditional projection-based screens. By optimizing the ratio of Yb^3+^ ions within the glass composition and employing techniques such as laser overlapping, we have shown the feasibility of producing controlled shapes within the glass volume. This study underscores the potential of glass material as a viable option for VDs, offering promising applications in true color imaging.

## Materials and methods

### Sample preparation

Selecting an appropriate glass matrix and the optimal amount of dopants to regulate energy transfers between RE^3+^ ions is a critical step in overcoming the complications that hinder the acquisition of a full-color laser-based VD screen material. Therefore, drawing from our previous experience^34–36^, glasses with the composition: 25Li_2_O-25WO_3_-47.65TeO_2_ + (0.05Ho_2_O_3_-0.25Tm_2_O_3_-0.05Nd_2_O_3_-2Yb_2_O_3_) mol% are selected, primarily due to the low phonon energy and high RE^3+^ solubility of this system, in addition to its numerous thermal, chemical, and mechanical advantages. The glass samples are fabricated via the melt-quenching method, employing high-purity chemicals including TeO_2_ (99.99%), WO_3_ (99.8%), Li_2_CO_3_ (99%), Ho_2_O_3_ (99.9%), Tm_2_O_3_ (99.9%), Nd_2_O_3_ (99.99%), and Yb_2_O_3_ (99.99%) to ensure the quality of the starting materials. A precise quantity of 50 g of the glass batch is meticulously weighed and homogenized by blending in an agate mortar. Subsequently, the mixture is transferred into a platinum crucible for the melting process, conducted in an electric furnace operating at a temperature of 800 °C for 30 min. Throughout the melting phase, continuous stirring is employed to achieve homogenous, highly transparent glass samples devoid of internal defects. Following the melting process, the molten glasses are poured into stainless-steel molds and promptly placed in a muffle furnace at 290 °C for 3 h for annealing. This annealing step is crucial for eliminating internal stresses within the glass structure. Finally, the glass samples undergo a natural cooling process and are subsequently polished to attain a high-quality surface suitable for optical characterizations. This comprehensive fabrication approach ensures the production of glass samples with optimal transparency and structural integrity, meeting the stringent requirements for 3D VD applications.

A thorough examination of the doping content of selected RE^3+^ ions in multi-doped systems is essential due to the numerous interactions between dopant ions, which can pose challenges in regulating energy transfer efficiency. Additionally, the relative intensities of primary colors (red, green, and blue) and their combinations (cyan, magenta, and yellow) are sensitive to various excitation parameters, such as frequency, pulse width, and power density, necessitating careful adjustments to achieve full-color tunability. Therefore, here we present an overview of the process involved in determining the optimal doping content of RE^3+^ ions in selected glass matrix. In our previous studies^27,28^, we generated multiple sample sets in the same tellurite base glass composition to explore optimal ratios of Ho^3+^, Tm^3+^, and Yb^3+^ ions and for the color tuning of upconversion via excitation modulation. Our observations revealed that the ideal combination of 0.05% Ho_2_O_3_, 0.25% Tm_2_O_3_ and 2% Yb_2_O_3_ facilitated the simultaneous emission of RGB light. However, blue emission was insufficient with Tm^3+^ ions alone in the glass structure. Herein, a strategic pairing of Nd^3+^ and Yb^3+^ ion, serving as sensitizer and activator ions, respectively, is reported to enable the utilization of a wide range of excitation wavelengths for optical population, significantly boosting the emission in three primary colors. Given the hypersensitivity of Nd^3+^ ions absorption to 808 nm excitation, a high concentration of Nd^3+^ ions that could significantly impact energy transfer to Yb^3+^ ions is undesirable^32,37^. Therefore, to enhance the blue emission, in this study, we additionally introduce a 0.05% ratio of Nd_2_O_3_ ions to the existing composition, striking a balance for increased blue emission while mitigating undesirable low-intensity transitions linked to higher concentrations.

### Laser path design and laser penetration depth investigation

To demonstrate the feasibility of our approach, a laser path is designed employing two distinct lasers, operating at wavelengths of 808 nm and 980 nm, respectively. This path is directed onto a galvanometer to create selected images within the glass sample. Focusing lenses are strategically placed along the laser path to ensure consistent laser beam diameters. The two laser beams are directed through a series of optical components, including mirrors, periscopes, focusing lenses, and a galvanometer, all positioned on the optical table, before being focused onto the glass sample. The operating frequencies and modulation parameters of the lasers are adjusted using a waveform generator. Additionally, a separate wavelength generator, operating through the EasyWaveX application, is used to control the independent movement of the galvanometer’s two mirrors along the laser path. The focal spot size of the scanning laser beam is determined using the formula derived from previous reports^8,18^: Spot Size = 1.83 × λ × f/A, where λ represents the laser wavelength, f denotes the focal length of the lens, and A indicates the diameter of the entrance beam. Additionally, the spot resolution is estimated to be approximately 100 μm × 100 μm (x × y). It is worth noting that further enhancement of resolution can be achieved by optimizing the alignment of the two laser inputs to reduce the focal volume of the scanning laser spot.

Before proceeding with image construction processes, we produced a range of glass samples with Yb_2_O_3_ doping concentrations ranging from 0.5% to 2% mol. This investigation aims to assess the influence of the Yb^3+^ ratio on the penetration depth of the laser within the glass sample, while keeping the base glass composition and the concentration of other RE^3+^ ions (RE = Ho, Tm, Nd) constant. It is widely recognized that higher concentrations of Yb^3+^ can lead to energy migration, resulting in energy loss due to quenching centers. Consequently, beyond a critical Yb_2_O_3_ doping concentration, non-radiative energy transfer occurs between Yb^3+^ and Nd^3+^ ions, a process commonly referred to as quenching^32^. Therefore, the Yb_2_O_3_ doping concentration is not increased beyond 2% mol. On the other hand, altering the Yb_2_O_3_ concentration is observed to have no effect on the radiative transitions and emission peak positions of other RE^3+^ ions. Instead, increasing the Yb_2_O_3_ concentration solely enhances the sensitivity of the 980 nm absorption band (Fig. S8a). It is observed that the penetration rate exhibits an inverse relationship with the Yb_2_O_3_ concentration in the glass, with images capable of forming at the center of the volume of a 5 mm-thick glass sample at a 2% mol Yb_2_O_3_ concentration. Consequently, at this Yb_2_O_3_ concentration square and hexagon images created on the sample surface diffuse into the sample, allowing the creation of 3D-like images (Fig. S8b). As the Yb_2_O_3_ concentration decreases, the distance that the laser can travel within the glass increases, with nearly 90% penetration achieved at a 1.5% Yb^3+^ concentration, while Yb_2_O_3_ concentrations of 1% or less allow the laser to pass entirely through the glass sample.

### Characterization studies

The Malvern PANalytical Empyrean Multicore High-Performance X-ray Diffractometer (XRD) with an average wavelength of Cu Kα1 and Cu Kα2 radiation (λ = 0.15418 nm, voltage: 45 kV, current: 40 mA) is employed to identify the amorphous nature of the as-cast samples in the 2θ range from 10° to 90° with a scanning step size of 0.02° (Fig. S9a). To assess the thermal behavior of the as-cast samples, thermal analysis measurements are conducted with a Netzsch STA 449 F3 Jupiter instrument. The glass transition (*T*_g_) temperature is determined by heating a selected powdered sample (30 ± 1 mg) in a platinum pan at a rate of 10 °C/min within a nitrogen environment (Fig. S9b). The optical absorption spectra of the samples are measured using an Edinburgh DS5 UV-Vis spectrophotometer with a resolution of 0.5 nm and a wavelength range of 190 to 1100 nm. PL measurements are conducted using an Edinburgh Instruments FS5 fluorescence spectrophotometer coupled with 980 nm and 808 nm lasers as the excitation sources. Time-resolved lifetime spectra are measured under excitation of 980 and 808 nm lasers using a time-correlated single-photon counting (TCSPC) method. The recorded decay curves are fitted using a bi-exponential equation to obtain the converging χ2 values to unity as indicated below^28^:

1 $$I\left(t\right)={A}_{1}{e}^{\left(\frac{-t}{{\tau }_{1}}\right)}+{A}_{2}{{\rm{e}}}^{\left(\frac{-t}{{\tau }_{2}}\right)}$$

where, $I\left(t\right)$ is emission intensity, ${\tau }_{1}$ and ${\tau }_{2}$ are the lifetime numerical values, and A_1_ and A_2_ are decay constants. Average lifetime of each transition is calculated by using the following equation^28^:

2 $${\tau }_{{meas}}=\frac{{A}_{1}{{\tau }_{1}}^{2}+\,{A}_{2}{{\tau }_{2}}^{2}}{{A}_{1}{\tau }_{1}+\,{A}_{2}{\tau }_{2}}$$

The rise time value is also calculated to determine the time constants, defining it as the duration required for a signal to transition from 10% to 90% of its final value. The OSRAM Color Calculator program is employed to calculate CIE (1931) color coordinates, based on the PL emission spectra of the samples in order to show the true color of the emissions.

## Supplementary information

Supplementary Information

Video S1

Video S2

Video S3

Video S4

Video S5

Video S6

## Data Availability

The data that support the findings of this study are available from the corresponding author upon reasonable request.
