# Supplementary material for: Full-color dynamic volumetric displays with tunable upconversion emission from RE3+-doped glasses (RE = Ho, Tm, Nd, Yb) under NIR laser excitation
Source: Light Sci Appl. 2025 Jan 2;14:15. doi: 10.1038/s41377-024-01672-2 (PMC11693759; doi:10.1038/s41377-024-01672-2)
Supplement: Supplementary file 1 — Supplementary Information [file 41377_2024_1672_MOESM1_ESM.docx]

Supplementary Information for:

**Full-Color Dynamic Volumetric Displays with Tunable Upconversion Emission from RE^3+^-Doped Glasses (RE = Ho, Tm, Nd, Yb) under NIR Laser Excitation**

Utku Ekim, Diğdem Özkutay, Miray Çelikbilek Ersundu*, Ali Erçin Ersundu*

Yildiz Technical University, Faculty of Chemical and Metallurgical Engineering, Department of Metallurgical and Materials Engineering, Glass Research and Development Laboratory, Istanbul, 34220, Türkiye

*Corresponding author‒E-mail: miray@yildiz.edu.tr (Miray Çelikbilek Ersundu), ersundu@yildiz.edu.tr (Ali Erçin Ersundu)

**
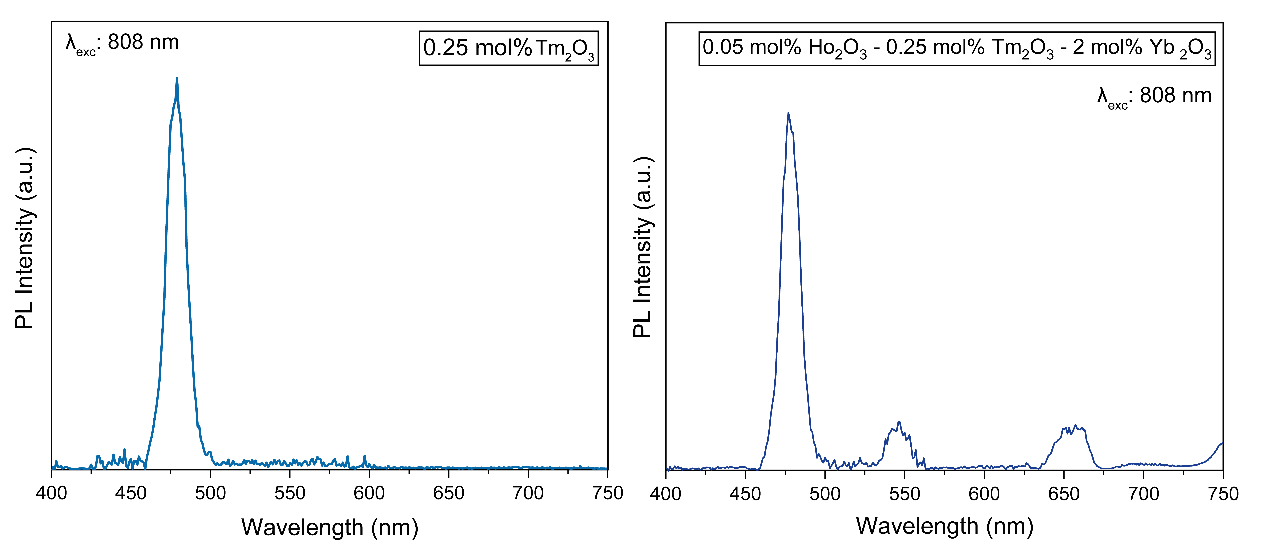
**

**Fig. S1** PL spectra of two additional RE^3+^ ions doped glasses, both without Nd_2_O_3_, under 808 nm excitation. a) PL spectrum of glass doped with 0.25 mol% Tm_2_O_3_, b) PL spectrum of glass doped with 0.05 mol% Ho_2_O_3_ - 0.25 mol% Tm_2_O_3_ - 2 mol% Yb_2_O_3_.

**Note:** a) The emission of glass doped with 0.25 mol% Tm_2_O_3_ demonstrates that Tm^3+^ can be directly excited at 808 nm, producing a prominent blue emission, indicating the presence of a direct upconversion pathway for Tm^3+^ under 808 nm excitation, b) The glass doped with 0.05 mol% Ho_2_O_3_ - 0.25 mol% Tm_2_O_3_ - 2 mol% Yb_2_O_3_ exhibits RGB emissions, attributed to energy transfer from Tm^3+^ to Yb^3+^, followed by Yb^3+^-mediated transfer to both Ho^3+^ and Tm^3+^. The red and green emissions are from Ho^3+^, while the blue emission is from Tm^3+^, confirming complex energy transfer dynamics under 808 nm excitation.

**
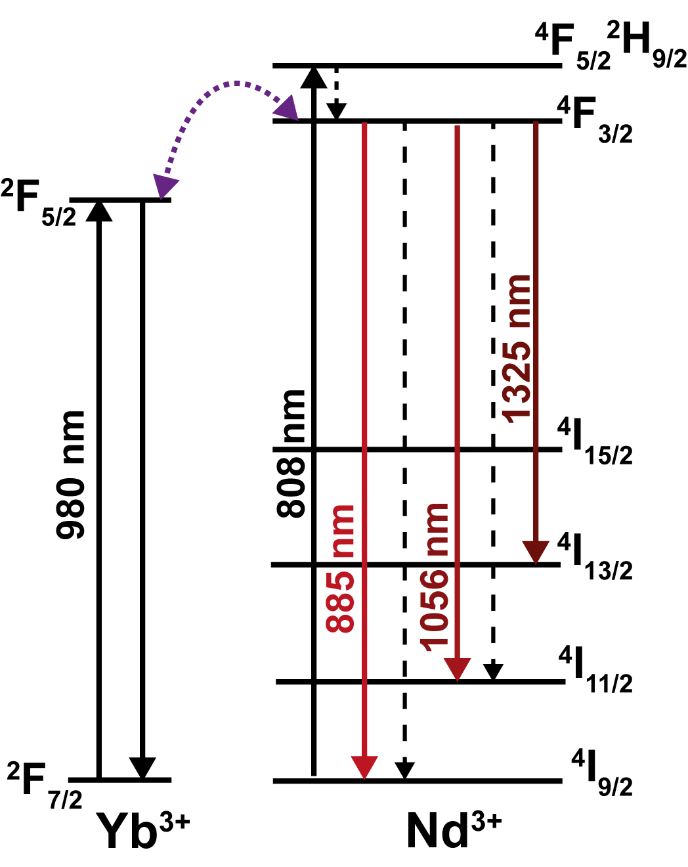
**

**Fig. S2** Partial energy level diagram of additional Yb^3+^ and Nd^3+^ ions-doped glass under 808 and 980 nm excitations.

**
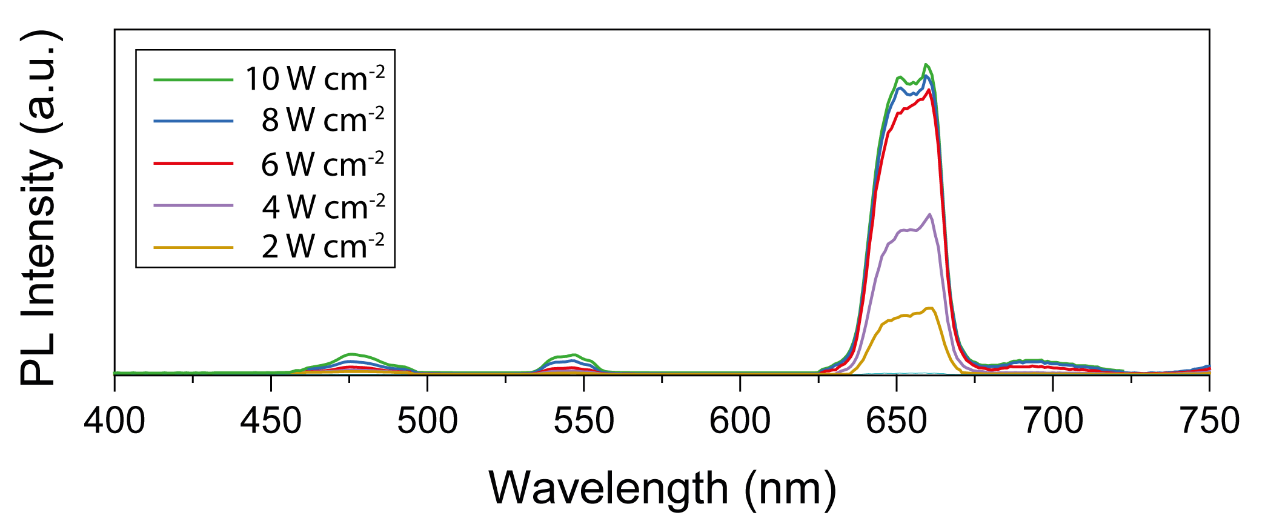
Fig. S3** PL emission spectra of RE^3+^-doped monolithic glasses (RE = Ho, Tm, Nd, Yb) under 980 nm laser excitation at varying power densities.

**Note:** The spectra demonstrate that red emission remains weaker at power densities below 6 W/cm^2^ but is significantly enhanced at 6 W cm^-2^. However, further increases in power density do not significantly alter the red emission intensity but lead to noticeable blue and green emissions, which hinders the acquisition of pure red emission. Consequently, a constant power density of 6 W cm^-2^ is used while adjusting excitation frequencies and pulse widths to achieve accurate RGB color reproduction.


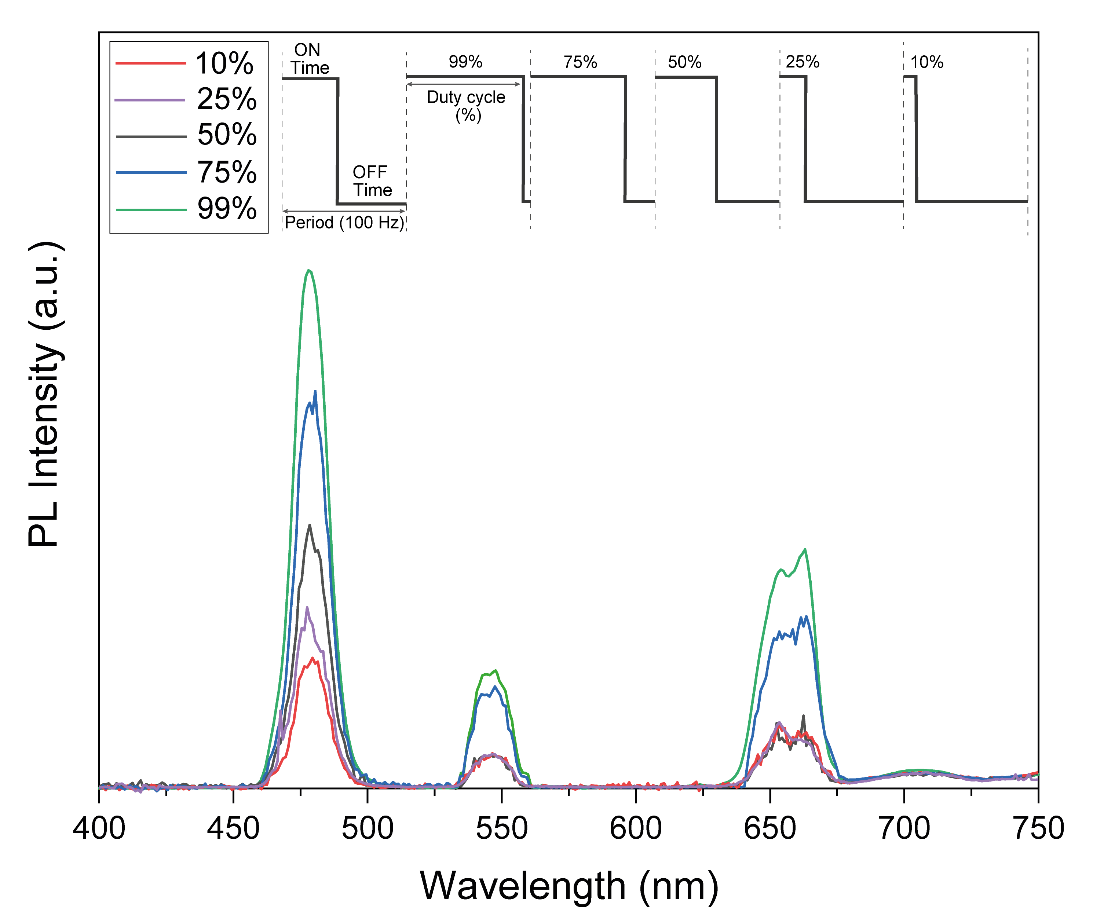


**Fig. S4** PL emission spectra excited by an 808 nm pulsed laser at a power density of 200 W cm^-2^ with varying duty cycles from 10 % to 99 %.

**Note:** Under 808 nm laser excitation, by switching to pulsed modulation, the blue emission intensity can be further controlled. Under a constant frequency of 100 Hz, increasing the duty cycle from 10 % to 99 % dominantly enhances the blue emission compared to the green and red emissions, demonstrating the ability to tune the emission color through duty cycle modulation.

**
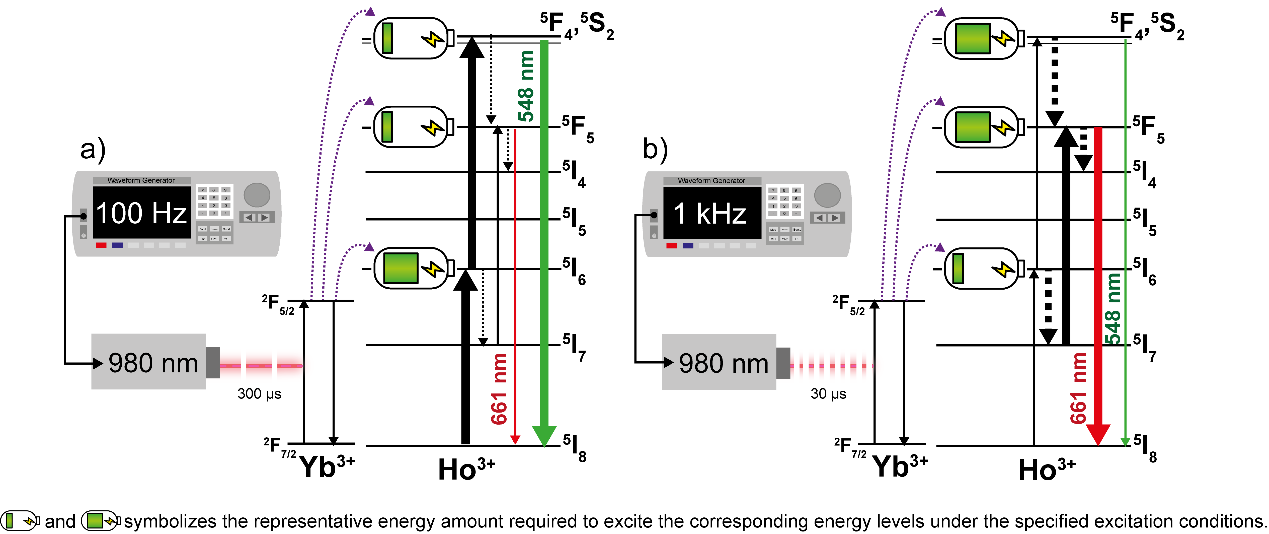
Fig. S5** An illustrative diagram of the time-dependent populating processes, visually representing the energy transfer dynamics for dominant green and red emissions.

**
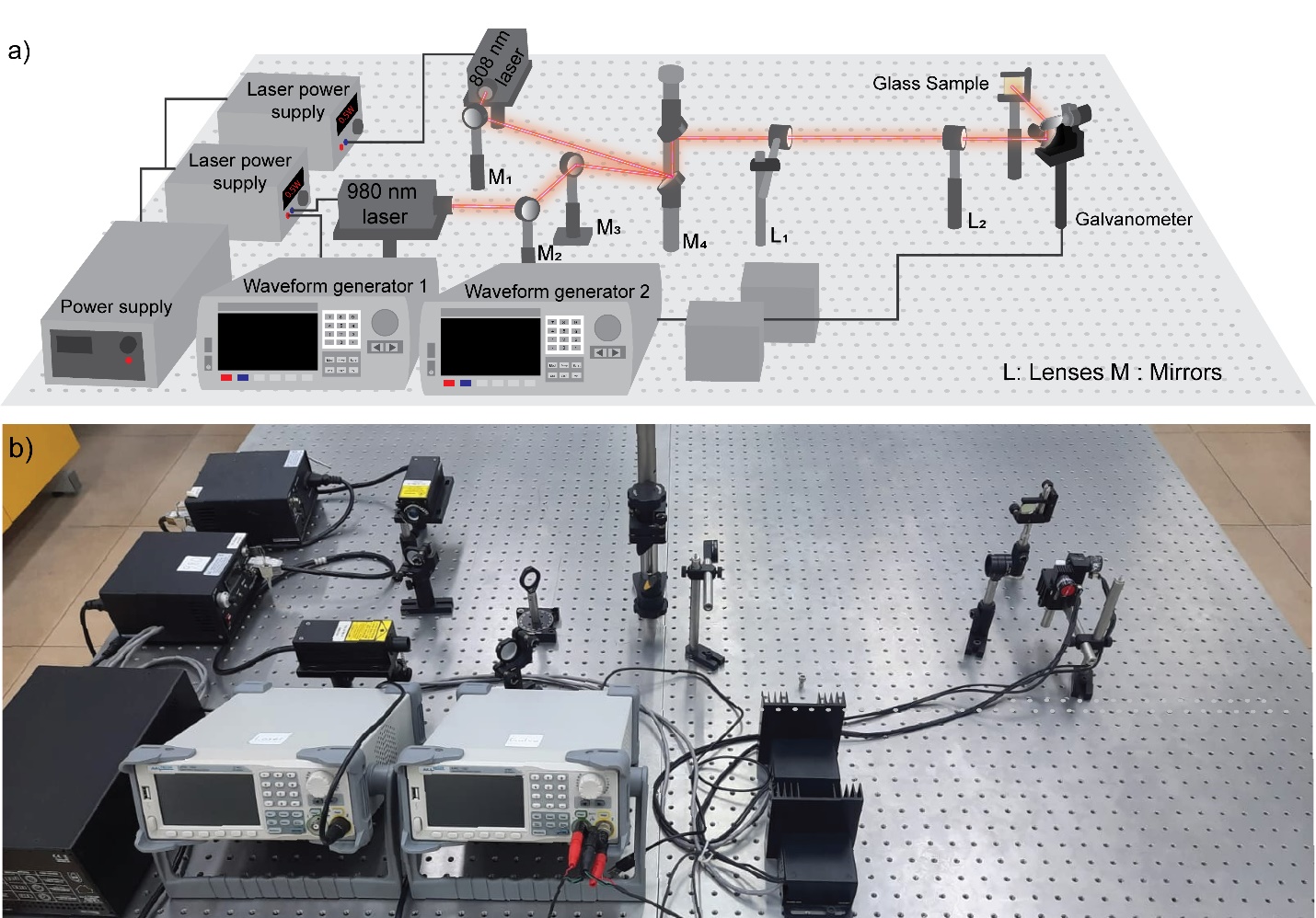
**

**Fig. S6** Schematic representation and top-view photograph of the designed setup for image construction.


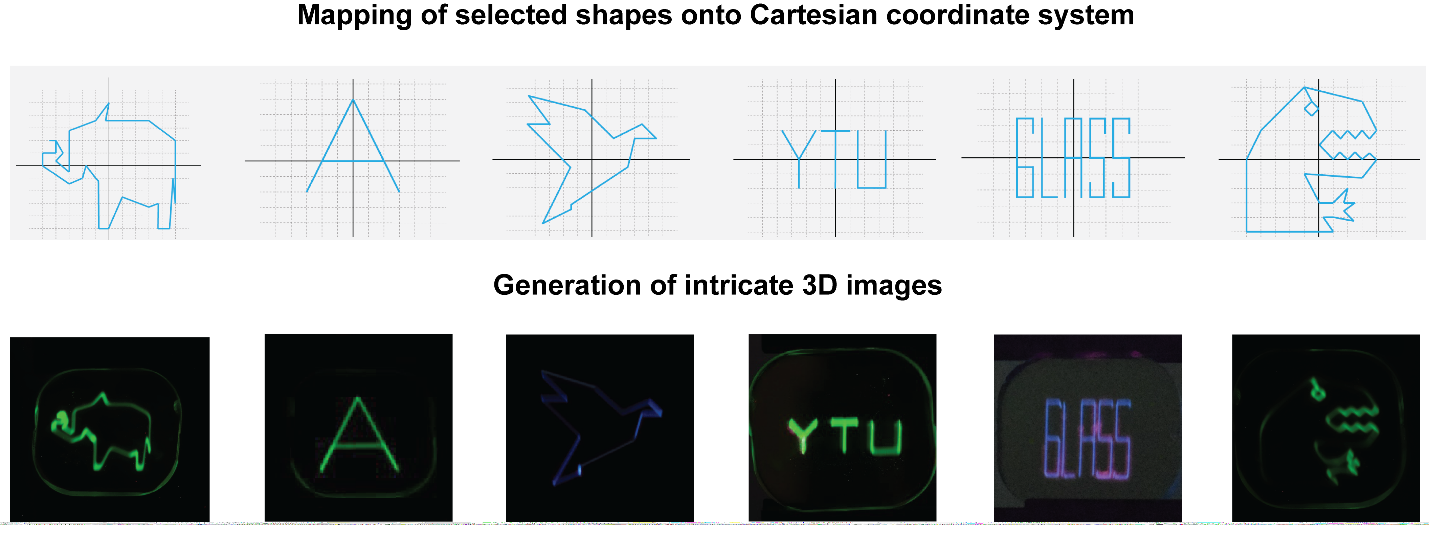
**Fig. S7** Generation of different intricate 3D images, ranging from basic geometric shapes to complex drawings.

**Note:** The non-uniformity in blue patterns is due to the difficulty of cameras in accurately detecting blue light and interference from the 808 nm laser beam, with residual inconsistencies despite using filters; future improvements with advanced cameras and lenses are anticipated to enhance uniformity and clarity.


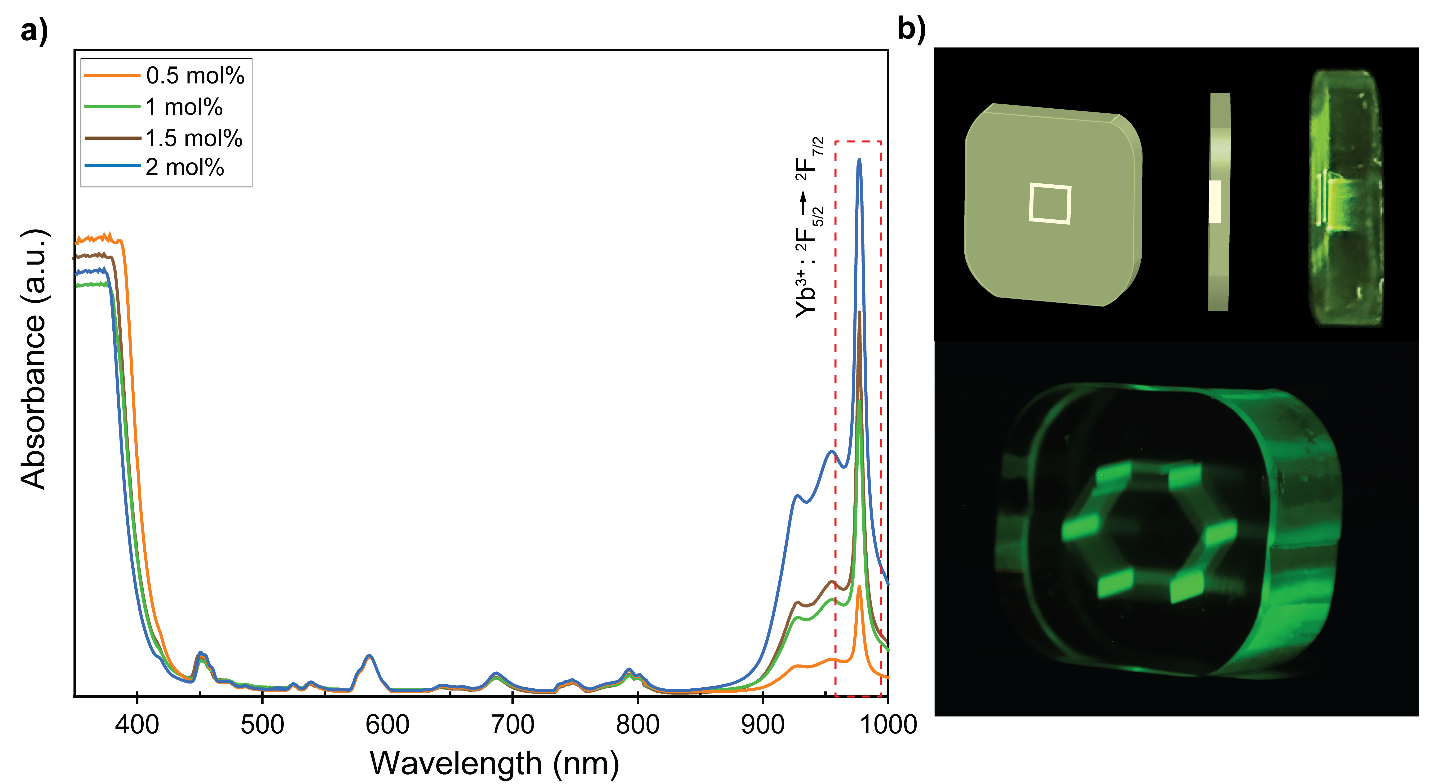


**Fig. S8** a) Optical absorption spectra of glass samples with Yb_2_O_3_ doping concentrations ranging from 0.5 mol% to 2 mol%. b) Penetration of a square image in the glass sample with 2 mol% Yb_2_O_3_ and 3D hexagon image fully diffusing into the glass.


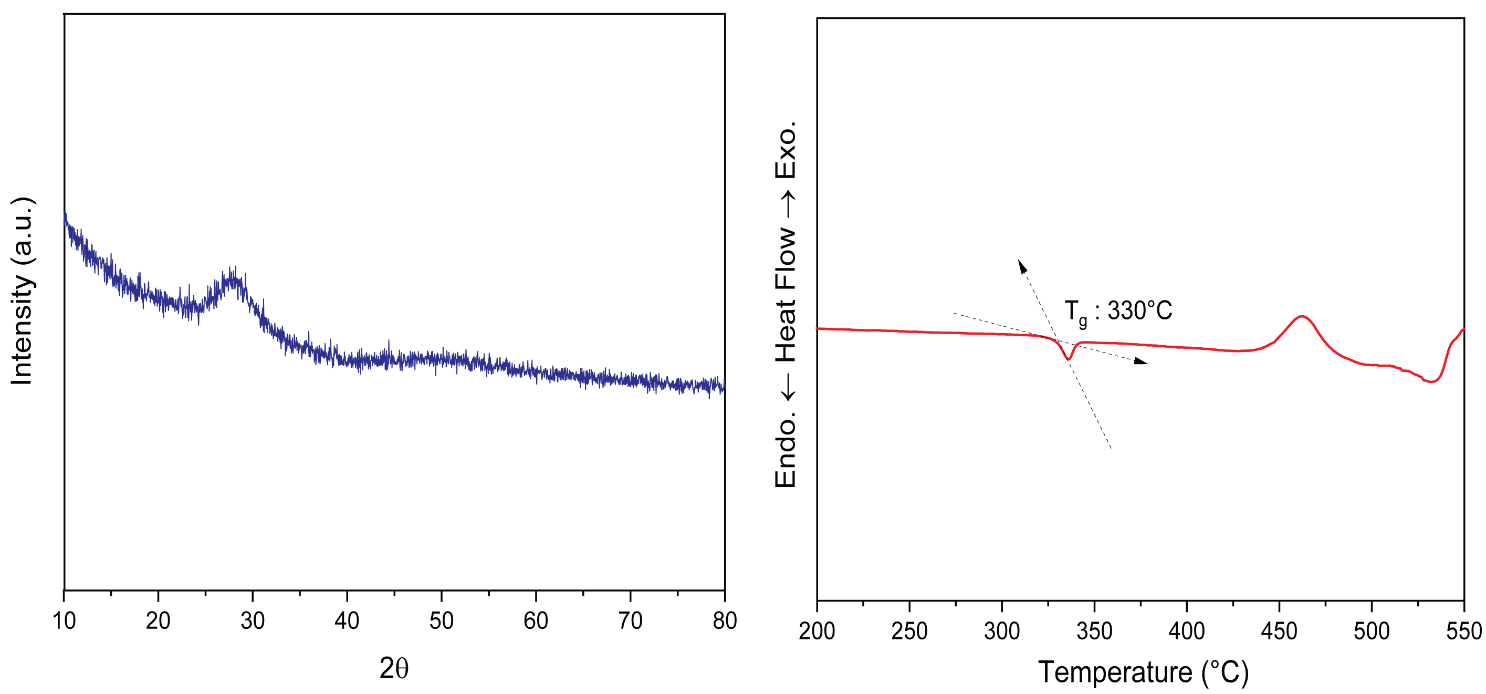


**Fig. S9** (a) XRD pattern, and (b) STA thermogram, depicting the thermal behavior and amorphous nature of the synthesized as-cast sample, respectively.

**Note:** The XRD analysis confirms the amorphous nature of the synthesized samples. In Figure S4(a), the XRD spectrum of the selected sample reveals a broad halo and the absence of distinct diffraction peaks, providing validation for its amorphous structure.

To establish appropriate annealing conditions, the thermal characteristics of the glass sample are investigated using simultaneous thermal analysis (STA). The STA curve for the selected sample is depicted in Figure S4(b), revealing a glass transition onset at 330 °C. Based on these results, a conservative annealing temperature of 290 °C, below the glass transition temperature (*T*_g_) to prevent crystallization, is chosen. The annealing process is conducted for 3 hours. The STA findings indicate that the synthesized glass sample exhibits resilience to high temperatures and heating-cooling cycles, crucial for maintaining structural integrity in laser-based display applications.

**Table S1** CIE color coordinates of RE^3+^ doped glasses under different

980, and 808 nm laser excitation.

| Excitation Conditions | Excitation Wavelength (nm) | CIE Color Coordinates (x, y) |
| --- | --- | --- |
| 1 | 980 | (0.58, 0.35) |
| 2 | 980 | (0.43, 0.39) |
| 3 | 980 | (0.49, 0.37) |
| 4 | 980 | (0.30, 0.65) |
| 5 | 980 | (0.27, 0.52) |
| 6 | 808 | (0.23, 0.26) |
| 7 | 808 | (0.17, 0.15) |
| 8 | 808 | (0.27, 0.35) |
